# Supplementary material for: EvoImp: Multiple Imputation of Multi-label Classification data with a genetic algorithm
Source: PLoS One. 2024 Jan 19;19(1):e0297147. doi: 10.1371/journal.pone.0297147 (PMC10798481; doi:10.1371/journal.pone.0297147)
Supplement: S1 Table — (PDF) [file pone.0297147.s001.pdf]

Table S1: Abbreviations

**Abbreviation**	**Description**
ACC	Accuracy
BR	Binary Relevance
CC	Classifier Chains
CMC	Concept Most Common
ECC	Ensembles of Classifier Chains
EM	Exact Match
GA	Genetic Algorithms
HL	Hamming loss
HOMER	Hierarchy of Multi-label classifiER
KDD	Knowledge Discovery in Databases
KMI	K-Means clustering Imputation
KNN	K-Nearest Neighbors
KNNI	K-Nearest Neighbors imputation
MC	Most Common
MCAR	Missing Completely At Random
MI	Multiple Imputations
ML-KNN	Multi-label K-Nearest Neighbors
MLC	Multi-Label Classifications
MV	Missing Values
NRMSE	Normalized Root-Mean-Square Error
ROC	Receiver Operating Characteristic
RMSE	Root-Mean-Square Error
SI	Single Imputation
SLC	Single-Label Classification
WKNNI	Weighted imputation with K-Nearest Neighbor
