# Supplementary material for: EvoImp: Multiple Imputation of Multi-label Classification data with a genetic algorithm
Source: PLoS One. 2024 Jan 19;19(1):e0297147. doi: 10.1371/journal.pone.0297147 (PMC10798481; doi:10.1371/journal.pone.0297147)
Supplement: Attachment — Submitted filename: PLOSONE - Response to Reviewers-2.pdf [file pone.0297147.s002.pdf]

RESPONSES TO REVIEWER 1

Reviewer #1: The authors describe Multiple Imputation of Multi-label classification data with a genetic algorithm, which is a very interesting study. The authors propose a novel imputation method based on genetic algorithm for optimizing multiple data imputations, and applies the proposed method in multi-label learning and evaluated its performance using six synthetic databases, considering various missing values distribution scenarios. But there is still room for improvement in the article

Point 1. Abstract: "Based on genetic algorithm" is too general to reflect the basic principles of the proposed method.

Response: Dear reviewer. Many thanks for providing us with the opportunity to again revise and resubmit our paper for consideration for PLOSONE. We reviewed the entire abstract and highlighted the following passage, which we believe clarifies the purpose of the method:

> “(...) One of the most usual missing data treatment methods is data imputation, which seeks plausible values to fill in the missing ones. In this scenario, we propose a novel imputation method based on a multi-objective genetic algorithm for optimizing multiple data imputations called EvoImp. We applied the proposed method in multi-label learning and evaluated its performance using six synthetic databases, considering various missing values distribution scenarios. The method was compared with other state-of-the-art imputation strategies.”

Point 2. Introduction: line16 The abbreviation MLOPs appears for the first time, please provide their full names

Response: Thank you for your careful review. Based on another reviewer's consideration, we removed the mentioned passage that required the acronym description (more details in Reviewer #2, point #2). Nevertheless, we conducted a rigorous review of all the acronyms used.

Point 3. In addition to the differences between the two classification tasks, I believe the author also needs to clarify the differences between single label data and multi label data, as well as why MultImp cannot or is not suitable for applying to multi label data, and then propose their own GAMultImp.

Response: Thank you for your valuable feedback. We appreciate your suggestion to clarify the differences between single label data and multi-label data in the

manuscript. To address this point, we have included a table that compares the two classification methods. The table provides a toy example with 5 instances and 3 labels, illustrating how the data is structured in each case. To facilitate the review process, we present below the excerpt that has been inserted into the section “Background”:

> “(...) Table 1 illustrates a toy example of the difference between SLC and MLC, adapted from (Tang et al. 2009). Considering that the data in Table 1 consists of 5 instances (x1, x2, x3, x4, x5) and 3 labels (y1, y2, y3).
>
> **Table 1.** Comparison of SLC and MLC using a toy example with 5 instances and 3 labels.
>
> | Data | Label |
> |---|---|
> | x_1 | y_1 |
> | x_2 | y_2 |
> | x_3 | y_3 |
> | x_4 | y_1 |
> | x_5 | y_3 |
>
> (a) Single-label
>
> | Data | Labels |
> |---|---|
> | x_1 | y_1, y_2 |
> | x_2 | y_2, y_3 |
> | x_3 | y_1, y_3 |
> | x_4 | y_2 |
> | x_5 | y_3 |
>
> (b) Multi-label
>
> The first subtable (Table 1a) illustrates the SLC scenario, where five data instances (x1 to x5) are each strictly associated with a single label (y1 to y3). For instance, x1 is associated with y1, x2 is associated with y2, and so on. On the other hand, MLC allows data instances to be associated with multiple labels simultaneously. The second subtable (Table 1b) demonstrates the MLC scenario, where the same five data instances (x1 to x5) can have multiple labels assigned to them. For example, x1 is associated with both y1 and y2, x2 is associated with both y2 and y3, and so forth. This distinction highlights how SLC restricts each data instance to a single label, while MLC permits instances to belong to multiple labels simultaneously, making it more suitable for scenarios where objects or data points may have multiple characteristics or attributes.”

Regarding why MultImp cannot or is not suitable for application to multi-label data and the rationale behind proposing a new method, it is essential to clarify the characteristics of both approaches.

The MultImp method was specifically designed for single-label classification, and its architecture has shown inefficiency when applied to multi-label classification tasks. This inefficiency primarily stems from challenges related to generating the initial population and the genotype-phenotype encoding process. Furthermore, MultImp relies on a multi-objective approach using Pareto frontier solutions. In summary, the individual population generation, genotype-phenotype encoding, and Pareto frontier-based solution make it unfeasible to apply the MultImp method in the multi-label classification scenario.

In contrast, the proposed method was explicitly designed to address multi-label classification challenges by reducing the search space as much as possible. This was achieved by employing simple imputation solutions as the initial population, varying the population size, and applying the mutation operator.

To avoid any misinterpretation of the method's names and to accurately reflect its nature, we have modified the nomenclature of the proposed method to EvoImp, combining the terms "Evolutionary" and "Imputation." As a consequence of this nomenclature change, we have updated the entire manuscript, as well the GitHub repository (https://github.com/jacobjr/evoimp), which contains the source code, test datasets, and supplementary materials.

We believe that these modifications and clarifications adequately address this point and provide a clearer understanding of the reasons behind proposing the EvoImp method for multi-label classification tasks.

Point 4. GAMultImp - Proposed Method. It is necessary to clarify whether the determination of the parameters of the crossover operator in line236 was determined through extensive experiments conducted in this article, or was it based on the results of experiments conducted by others. Is the candidate value exchanged by the line240 mutation operator computationally generated, randomly generated, or self-defined. Exact explanation is required.

Response: Thank you very much for your observation. It is indeed necessary to clarify the process of determining the crossover parameters. In this regard, we conducted initial experiments to find the optimal configuration of these parameters. To provide further details, we have included the following excerpt in the manuscript, which contains the references used and the guidance received from them.

> “In the literature, numerous methods for parameter tuning and control have been proposed and analyzed. [41] provide descriptions of some of these methods and discuss various trends and challenges in the field. Specifically, [42] conducted experiments to find appropriate settings for these parameters in the context of applying evolutionary algorithms to a multi-objective problem class. They concluded that determining the value of the scaling factor can be difficult and is highly dependent on the specific problem. Considering these findings, initial tests were conducted to define the parameters used in our study. In line with the work of [42], the initial percentage of Crossover was delimited to [0.8, 1.0], following the common proposal for non-separable problems like the one tackled in our research. EvoImp employs a crossover for 80% of the individuals using an n-point crossover operator [40]. It is also consonant with the work of [36].”

Regarding the candidate values used in the mutation operator, it is important to clarify that these values are computationally generated. The candidate values are formed by a set of possible values (representing all the possibilities for a specific attribute in the evaluated dataset). In this context, for a better understanding, we have included the following excerpt and figure in the manuscript:

> "(...) each attribute in the dataset has a set of solutions, as shown in Table 2. This set is formed by considering all possible response options for that attribute in the evaluated dataset.
>
> **Table 2.** Example of the representation of the sets of candidate solutions for each attribute, used for the mutation process.
>
> | Year | Gender | Age | Have Credit |
> |---|---|---|---|
> | 2010 | M | 25 | ? |
> | ? | F | ? | Yes |
> | 2005 | ? | 32 | ? |
> | 1998 | M | ? | Yes |
> | ? | ? | 30 | No |
>
> (a) Toy Example of a dataset with MV.
>
> | Attribute | Values |
> |---|---|
> | Year | 1998, 2005, 2010 |
> | Gender | M, F |
> | Age | 25, 30, 32 |
> | Have Credit | Yes, No |
>
> (b) Set of possible values
>
> Table 2a displays a toy dataset containing five records and four attributes: ”Year,” ”Gender,” ”Age,” and ”Have Credit.” Some values in the dataset are missing and are represented by ”?”. The table 2b lists the possible values for each attribute. For example, the ”Year” attribute can have values 1998, 2005 or 2010, the ”Gender” attribute can have values M or F, and so forth.”

Once again, we express our sincere gratitude for your meticulous review of our manuscript. We hope that our actions fulfill your expectations.

RESPONSES TO REVIEWER 2

Reviewer #2. This manuscript uses genetic algorithms to solve the multiple imputations problem in multi-label learning. The experiments are adequate and the methods are analyzed and discussed. However, the manuscript still suffers from the following problems.

Point 1. The abstract is too verbose about the background and the paper's focus and innovation are unclear.

Response: Dear reviewer. We sincerely appreciate the opportunity to revise and resubmit our paper for further consideration at PlosOne. Thank you for your valuable feedback and suggestions.

We performed a general revision of the abstract. In this regard, we removed a part of the context focusing on the main problem (missing data and multi-label classification) and added more information about the work developed.

To facilitate the review process, we present below the new abstract:

> "Missing data is a prevalent problem that requires attention, as most data analysis techniques are unable to handle it, compromising the reliability of results. Despite its significance, only a few studies have investigated Multi-label Classifications (MLC) with missing data. Multi-label learning is considered an emerging and promising research topic because of the growing number of new applications, such as the semantic classification of videos and images, music categorization, and medical diagnostics. One of the most usual missing data treatment methods is data imputation, which seeks plausible values to fill in the missing ones. In this scenario, we propose a novel imputation method based on a multi-objective genetic algorithm for optimizing multiple data imputations called EvoImp. We applied the proposed method in multi-label learning and evaluated its performance using six synthetic databases, considering various missing values distribution scenarios. The method was compared with other state-of-the-art imputation strategies. The results proved that the proposed method outperformed the baseline in all the scenarios, representing a feasible solution to missing data treatment for multi-label learning. Following open-science principles, the source codes and datasets are publicly available in a GitHub repository."

Point 2. The first two introductory paragraphs in Introduction Section are confusing and it is impossible to understand what the author is trying to convey.

Response: Thank you very much for your observation. Indeed, the text was confusing. In this regard, just like we did in the abstract, we reduced the contextualization by completely removing the first paragraph and a significant portion of the second.
To facilitate the review process, we present how the first two paragraphs of the Introduction section were revised:

> “One of the most time-consuming tasks in the data mining pipeline is related to data preparation. There is a consensus in this field that data preparation is responsible for up to 80% of the entire process of discovering information [1]. In this case, selecting the dataset is one of the first steps in the process and can help reduce the effort required in the preprocessing phase [2]. In practice, real-world datasets contain a proportion of instances with Missing Values (MVs) caused by various factors surrounding the data acquisition system [3]. Unless they are handled properly, these absences can harm the result’s reliability, mainly when the MVs distribution is not uniform, and the mechanism that might cause these missing values is unknown [4].
>
> Several techniques have emerged to address this problem. LIN [2] comments that if the MVs rate is less than 10% or 15%, they can be removed without causing any significant loss to the mining process. However, this does not mean that the datasets in any problem domain must follow this rule; in other words, small amounts of missing data may contain essential information that must be managed [5]. Another approach that is quite widespread in the literature and non-trivial is the use of data imputation methods that can be employed to search for what is regarded as plausible values to fill any gaps that might be found [4, 6–8].”

With these modifications, we hope the text has become clearer and more fluent.

Point 3. Please redraw the Algorithm 1 flowchart.

Response: Thank you very much for your contribution. We have provided the flowchart of the algorithm in the repository as supplementary material (https://github.com/jacobjr/EvoImp). To facilitate the review process, we present the flowchart below:

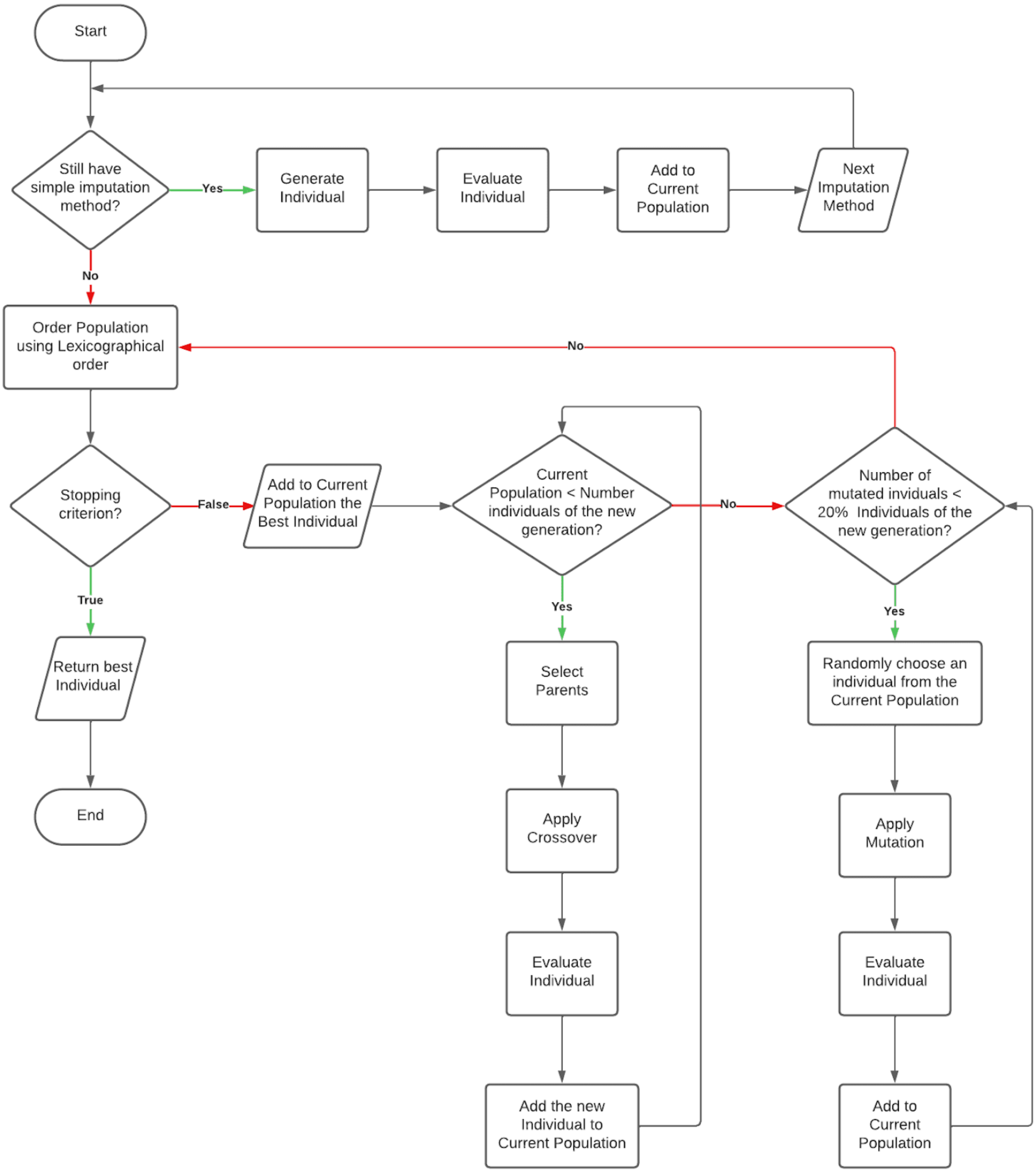

Fig. 1. Algorithm flowchart.

Additionally, we made efforts to improve the algorithm presented in the manuscript. To achieve this, we sought examples of algorithms from previously published works in PlosOne. Specifically, we highlighted the following articles:

1. Wu J, Zhang Z, Tong R, Zhou Y, Hu Z, Liu K (2023) Imaging feature-based clustering of financial time series. PLoS ONE 18(7): e0288836. https://doi.org/10.1371/journal.pone.0288836

2. Xu Y, Yu X, Wang T, Xu Z (2020) Pooling region learning of visual word for image classification using a bag-of-visual-words model. PLoS ONE 15(6): e0234144. https://doi.org/10.1371/journal.pone.0234144

Based on this research, we updated the algorithm presented in the manuscript. To facilitate the review process, we provide both the previous version and the new version of the updated algorithm:

Algorithm 1: GAMultImp

```
Data: database with MV, simple imputation methods, sizes of the generations
Result: complete database
foreach simple imputation method do
    individual ← generateIndividual(method, databaseWithMV);
    evaluateInd(individual, [EM, ACC, HL]);
    currentPop ← add(individual);
end
population ← orderByLexicography(currentPop, [EM, ACC, HL]);
foreach generation do
    newPopulation ← population;
    currentPop ← bestIndividualInTheNewPopulation;
    while currentPop ≤ generation do
        parent1 ← selectParent(newPopulation);
        parent2 ← selectParent(newPopulation);
        newIndividual ← crossover(parent1, parent2);
        evaluateInd(newIndividual, [EM, ACC, HL]);
        currentPop ← add(newIndividual);
    end
    while numberOfMutations ≤ generation * 0.2 do
        individualChosen ← random(currentPop);
        applyMutation(individualChosen);
        evaluateInd(individualChosen, [EM, ACC, HL]);
        currentPop ← add(individualChosen);
    end
    population ← orderByLexicography(currentPop, [EM, ACC, HL]);
end
return bestIndividualOfPopulation;
```

Fig. 2. Old Algorithm.

Algorithm 1: EvoImp

```
Input: database with MV and parameters (see Table 4)
Output: complete database
foreach Simple Imputation Method do
    Generate a new Individual: individual;
    Evaluate the individual;
    Add individual to the Current Population: currentPop ← individual;
end
Order currentPop using Lexicographical order;
while Stop criterion not reached do
    Add to the Current population the Best Individual: currentPop ← bestIndividual;
    while currentPop < Number individuals of the new generation do
        Select Parents;
        Appy Crossover;
        Evaluate the new Individual;
        Add the Individual to Current population: currentPop ← individual;
    end
    while Number of mutated individuals < 20% of Individuals of the new generation do
        Randomly choose an individual from the Current population;
        Apply Mutation;
        Evaluate the Individual;
        Add the Individual to Current population: currentPop ← individual;
    end
    Order currentPop using Lexicographical order;
end
return bestIndividual;
```

Fig. 3. New version of the Algorithm.

Point 4. What do the '16 scenario databases' mentioned in Binary Relevance in Results and Analysis mean? If it refers to the number of datasets that performed well in terms of accuracy, the results shown in Table 3 should be 18.

Response: Thank you for your careful reading. Indeed, the value was incorrect. To provide a clearer result and make the correction, the new passage in the manuscript is:

> "The method demonstrated superior performance compared to others in 18 scenario databases (50%) as evaluated using the accuracy of the BR classifier."

Furthermore, we took the opportunity to review all the results and found other incorrect values, which have been rectified in the new version of the manuscript and are indicated in the highlighted version.

We extend our utmost gratitude once again for your diligent review of our manuscript. Your insightful evaluation and feedback have played a pivotal role in refining our work. We earnestly acknowledge the time and effort you devoted to this task. We have strived to comprehensively address all the concerns you raised and to ensure their relevance and clarity in our revisions.
