# Supplementary material for: EvoImp: Multiple Imputation of Multi-label Classification data with a genetic algorithm
Source: PLoS One. 2024 Jan 19;19(1):e0297147. doi: 10.1371/journal.pone.0297147 (PMC10798481; doi:10.1371/journal.pone.0297147)
Supplement: Attachment — Submitted filename: PLOSONE - Response to Reviewers - v2023-11.pdf [file pone.0297147.s004.pdf]

Manuscript No. PONE-D-23-16570R1
Title: “EvoImp: Multiple Imputation of Multi-label classification data with a genetic algorithm”

Dear Editor,

We are grateful for the opportunity to revise and resubmit our paper. We have made sincere efforts to address all suggestions.

Following, we present detailed responses to the comments you and the two reviewers provided. The original comments are highlighted in bold, while our responses are presented in standard text.

We hope you find the quality of this paper has been improved.

Kind regards,
The Authors

<u>**RESPONSES TO EDITOR**</u>

ED #1: Abstract - Remove “, compromising the reliability of results. ” from second line

Dear Editor, many thanks for allowing us to revise and resubmit our paper for consideration for PLOSONE. This excerpt was removed.

ED #2: As authors are focusing on MLC, the first sentence should state why addressing missing values in MLC is important rather starting it from general perspective of missing value.

Thank you for the careful reading. We rewrote this part, making the MLC more evident.

ED #3: Define what is MLC in the abstract for the multidisciplinary readers of the journal

Thank you for this observation. We have defined MLC, providing an illustrative example, considering the multidisciplinary readership of the journal. To facilitate the review process, we provide the excerpt below:

> “(...) This is particularly critical in Multi-Label Classification (MLC), where only a few studies have investigated missing data in this application domain. MLC differs from Single-Label Classification (SLC) by allowing an instance to be associated with multiple classes. Movie classification is a didactic example since it can be ``drama'' and ``bibliography'' simultaneously. (...)”

ED #4: This sentence is redundant to the focus of the manuscript “Multi-label learning is considered an emerging and promising research topic because of the growing number of new applications, such as the semantic classification of videos and images, music categorization, and medical diagnostics.” Remove it

Thank you for the careful reading of our work. We agree that this part is redundant. It has been removed.

ED #5: Use the full form for “EvoImp” before using it for example Evolutionary Imputation method (EvoImp)

Thank you for this suggestion. We have revised the manuscript following your recommendation, and the excerpt is now:

> “(...) In this scenario, we propose a novel imputation method based on a multi-objective genetic algorithm for optimizing multiple data imputations called Multiple Imputation of Multi-label Classification data with a genetic algorithm, or simply EvoImp. (...)”

ED #6: Add real results how EvoImp outperformed other methods and why it is innovative - The method was compared with other state-of-the-art imputation strategies….. such as (add some names)

Thank you for this pertinent suggestion. We have described how EvoImp outperformed the other methods by explicitly addressing the following aspects: i) the baseline (state-of-the-art imputation strategies), ii) the evaluation measures (Exact Match, Accuracy, and Hamming Loss), and iii) complementary experimental setup (dataset characteristics). Here we provide excerpt with these improvements:

> “(...) We applied the proposed method in multi-label learning and evaluated its performance using six synthetic databases, considering various missing values distribution scenarios. The method was compared with other state-of-the-art imputation strategies, such as K-Means Imputation (KMI) and weighted K-Nearest Neighbors Imputation (WKNNI). The results proved that the proposed method outperformed the baseline in all the scenarios by achieving the best evaluation measures considering the Exact Match, Accuracy, and Hamming Loss. The superior results were constant in different dataset domains and sizes, demonstrating the EvoImp robustness. (...)”

ED #7: Remove this “Following open-science principles, the source codes and datasets are publicly available in a GitHub repository.”

Thank you for your suggestion. This part has been removed from the abstract.

ED #8: Introduction. Remove these lines, One of the most time-consuming tasks in the data mining pipeline is related to data 2 preparation. There is a consensus in this field that data preparation is responsible for 3 up to 80% of the entire process of discovering information [1]. In this case, selecting the 4 dataset is one of the first steps in the process and can help reduce the effort required in 5 the preprocessing phase [2].

Thank you for your relevant suggestion. We have removed the indicated excerpts and the bibliographic references associated with them.

ED #9: The author should start their introduction section with how missing values impact the scientific studies, decision making etc.

Thank you for your observation. We have revised the introduction to address this concern, also incorporating relevant references. To facilitate the review process, we provide the excerpt and the references as follows:

> “Missing data is ubiquitous in data analysis [1]. Their causes are the most diverse and related to the application domain. These include drawbacks in data acquisition, measurement errors, sensor network problems, data migration failures, and unwillingness to respond to survey questions [2, 3]. Since data analysis algorithms/methods are not designed to deal with

Missing Values (MVs), it is essential to treat them before aiming to guarantee the results' validity, impairing the research conclusions [1, 4, 5]. MVs are problematic because of the risk of bias, which depends on the type of missing data, the extent of the missingness, and how to deal with MVs in the analyses [1]. Thus, it is critical to deal with the missing data timely for intelligent decision-making [6]."

References

[1] Heymans MW, Twisk JW. Handling missing data in clinical research.. Journal of clinical epidemiology. 2022 Nov 1;151:185.

[2] Honaker J, King G. What to Do about Missing Values in Time-Series Cross-Section Data. American Journal of Political Science. 2010 Apr 54;2:561-581

[3] Tsai CF, Li ML, Lin WC. A class center based approach for missing value imputation. Knowledge-Based Systems. 2018 Jul 151:124-35. DOI:10.1016/j.knosys.2018.03.026.

[4] Lin WC, Tsai CF. Missing value imputation: a review and analysis of the literature (2006–2017). Artificial Intelligence Review. 2020 Feb;53:1487-509.

[5] Garciarena U, Santana R. An extensive analysis of the interaction between missing data types, imputation methods, and supervised classifiers. Expert Systems with Applications. 2017 Dec 15;89:52-65. DOI:10.1016/j.eswa.2017.07.026

[6] Adhikari D, Jiang W, Zhan J, He Z, Rawat DB, Aickelin U, Khorshidi HA. A comprehensive survey on imputation of missing data in internet of things. ACM Computing Surveys. 2022 Dec 15;55(7):1-38.

ED #10: Considering changing this sentence "Several techniques have emerged to address this problem. ", as authors did not provided many examples here

Thank you for carefully reviewing our work. We acknowledge that this part lacked a solid theoretical basis. Nevertheless, we firmly believe that this assertion is relevant to our research. To substantiate this claim, we have included references to some seminal works, reviews, and surveys that summarize these methods, as follows:

[4] Lin WC, Tsai CF. Missing value imputation: a review and analysis of the literature (2006–2017). Artificial Intelligence Review. 2020 Feb;53:1487-509.

[7] Luengo J, Garcia S, Herrera F. On the choice of the best imputation methods for missing values considering three groups of classification methods. Knowl Inf Syst. 2012;32(1):77–108.

[8] Emmanuel T, Maupong T, Mpoeleng D, Semong T, Mphago B, Tabona O. A survey on missing data in machine learning. Journal of Big Data. 2021 Dec;8(1):1-37. DOI: 10.1186/s40537 − 021 − 00516 − 9.

ED #11: Line 15-17 Revise this sentence, the current sentence does not make any sense ….Another approach 15 that is quite widespread in the literature and non-trivial is the use of data imputation 16 methods that can be employed to search for what is regarded as plausible values to fill 17 any gaps that might be found [4,6–8].

Thank you for your valuable feedback. We have addressed your comment regarding revising the sentence in lines 15-17. The original sentence was indeed unclear. The revised text now reads as follows:

> "In addressing this issue, the literature suggests using missing data imputation methods, which involve replacing missing data with actual (plausible) values. While this approach allows for more data retention compared to deletion, it requires time to generate reasonable replacement values [10, 11]."

References

[10] Ren L, Wang T, Seklouli AS, Zhang H, Bouras A. A review on missing values for main challenges and methods. Information Systems. 2023 Oct 119. DOI:10.1016/j.is.2023.102268

[11] Farhangfar A, Kurgan L, Dy J. Impact of imputation of missing values on classification error for discrete data. Pattern Recognition. 2008 Dec 1;41(12):3692-705

ED #12: Line 20, Define what is Multiple Imputations (MI) before moving forward to more advanced imputation methods

Thank you for your helpful suggestion. We understand the importance of providing clear definitions for the imputation methods discussed in our work. In response to your comment, we have introduced the concept of Single Imputation (SI) and its characteristics as follows:

> "A naïve method for tackling the missing values issue is by Single Imputation (SI). This method involves filling in missing values with a single estimated value, often based on mean, median, or regression models [4]. While this approach simplifies the dataset and makes it easier to analyze, it can introduce bias and underestimate uncertainty in the results [12, 13]. To overcome this limitation, Rubin [14] introduced a gold-standard imputation strategy within the scientific community - Multiple Imputations (MI) for handling missing data. In contrast with SI approaches, this method seeks to find a single solution in which m complete solutions are created in the operational database such that m > 1. These solutions were analyzed separately and combined to obtain the best solution [15, 16]. To reduce the missing values prediction error, using metaheuristics could optimize the value that would be imputed [15]. Notably, bioinspired strategies such as Genetic Algorithms (GAs) are prominent in optimizing solutions [17]."

References

[4] Lin WC, Tsai CF. Missing value imputation: a review and analysis of the literature (2006–2017). Artificial Intelligence Review. 2020 Feb;53:1487-509

[12] Rubin DB. An overview of multiple imputation. In: Proceedings of the survey research methods section of the American statistical association 1988 Aug (Vol. 79, p. 84). Princeton, NJ, USA: Citeseer.

[13] Li P, Stuart EA, Allison DB. Multiple imputation: a flexible tool for handling missing data. Jama. 2015 Nov 10;314(18):1966-7

[14] Rubin DB. Multiple imputation for nonresponse in surveys. John Wiley & Sons; 2004 Jun 9.

[15] Lobato FMF. Evolutionary strategies to optimize the treatment of missing data by multiple imputation data (in Portuguese). PhD Thesis, Federal University of Pará, 2016.

[16] Nunes LN, Kluck MM, Fachel JMG. Use of multiple imputation for missing data: a simulation using epidemiological data (in Portuguese). Cad Saúde Pública [online]. 2009;25(2):268-278. DOI: 10.1590/S0102 − 311X2009000200005.

[17] Chiu PC, Selamat A, Krejcar O, Kuok KK, Bujang SD, Fujita H. Missing Value Imputation Designs and Methods of Nature-Inspired Metaheuristic Techniques: A Systematic Review. IEEE Access. 2022 May 9.(pp. 61544-61566) DOI:10.1109/ACCESS.2022.3172319

ED #13: Line 30, “MultImp algorithm is coming out of nowhere, define it”

Thank you for your valuable feedback. To provide more context and clarity, we have included a definition and introduced the algorithm with a reference to its source. Furthermore, we have added a new reference to support this introduction and have revised the sentence to read as follows:

> “The GAs were proposed by Holland [18]. It is an optimization heuristic based on “the survival of the fittest”, inspired by Charles Darwin's evolutionary theory. Regarding the GAs usage for Multiple Imputations, it is crucial to acknowledge the work of Garcia [19] and the MultImp algorithm [15]. The MultImp algorithm serves as the cornerstone for this research. This algorithm employed genetic algorithms for multiple imputations and was also applied for Multi-Label Classification (MLC) scenarios. The authors contend that data mining tasks, particularly those related to data classification, are notably sensitive to addressing MV. Furthermore, classification tasks are widely used to assess the accuracy (ACC) of imputation methods [5, 11, 20]. Consequently, the higher the classification accuracy, the more successful the imputation method. However, only a few studies have employed MLC. In contrast to Single-Label Classification (SLC), or simply data classification, which associates an example with a single label, MLC allows an instance to be associated with multiple labels, thereby increasing the complexity of classification tasks [21, 22]. Further details on this topic will be highlighted in the Background section.”

References

[5] Garciarena U, Santana R. An extensive analysis of the interaction between missing data types, imputation methods, and supervised classifiers. Expert Systems with Applications. 2017 Dec 15;89:52-65. DOI:10.1016/j.eswa.2017.07.026.

[11] Farhangfar A, Kurgan L, Dy J. Impact of imputation of missing values on classification error for discrete data. Pattern Recognition. 2008 Dec 1;41(12):3692-705.

[15] Lobato FMF. Evolutionary strategies to optimize the treatment of missing data by multiple imputation data (in Portuguese). PhD Thesis, Federal University of Pará, 2016.

[18] Holland JH. Adaptation in natural and artificial systems: an introductory analysis with applications to biology, control, and artificial intelligence. MIT press; 1992 Apr 29.

[19] Garcia JCF, Kalenatic D, Bello CAL Missing data imputation in multivariate data by evolutionary algorithms. Comput Hum Behav. 2011. 27:1468–1474.

[20] Provost F, Saar-Tsechanski M. Handling Missing Values when Applying Classification Models. Journal of Machine Learning Research. 2007;8.

[21] Read J, Pfahringer B, Holmes G, Frank E. Classifier chains for multi-label classification. Machine learning. 2011 Dec;85:333-59.

[22] Ghani MU, Rafi M, Tahir MA. Discriminative adaptive sets for multi-label classification. IEEE Access. 2020 Dec 1;8:227579-95. DOI:10.1109/ACCESS.2020.3041763.

ED #14: Line 28-34: I suggest authors to define what is MLC, how GA-based imputation would have been used previously, what are the drawbacks of prior algorithms?

Thank you once again for your valuable feedback. We appreciate your input, and with the previous changes, we believe that the issue raised has been effectively addressed. Furthermore, we would like to highlight that we have a discussion of the difference between Single-label and Multi-label Classification in Section 2 (Background) of our paper. We hope this serves to provide a more comprehensive understanding of the concepts presented in our research.

ED #15: Line 36-37: remove the sentence

Thank you for your helpful suggestion. This excerpt was removed.

ED #16: Line 47-49: Revise the sentence, the current format dose not make any sense. Also, the name refers to MultImp [12], on which the 47 algorithm was based, since it is a strategy that achieves promising results (although it is 48 still in the preliminary stage) concerning multiple imputations for missing data

Thank you for your constructive feedback. We have taken your comments into account and have revised the paragraph in question:

> “Considering the importance of handling missing values in data analysis and the available solutions in the existing literature, this work presents an efficient

> algorithmic approach for multiple imputations applied to multi-label classification tasks. This method is named EvoImp, a combination of “evolutionary” and “imputation”. Furthermore, the name is inspired by MultImp [15], which serves as the foundation for our algorithm and has shown promise in its preliminary stages for multiple imputations with missing data. EvoImp enhances the parameterization of MultImp to maximize its imputation capabilities and explores new configurations for computational experiments.”

ED #17: Author should add a paragraph talking about the drawbacks/limitation of the some existing MLC imputation methods, then start the methodology

We sincerely appreciate the insightful comment. In our recent updates to the paper, specifically in the section where we discuss the distinctions between Single and Multiple imputation, we have effectively addressed the limitations of imputation methods. Moreover, we have added a paragraph explaining the methodology, serving to conclude the Introduction section as follows:

> “We conducted a rigorous benchmarking process to validate the proposed method's performance using diverse multi-label datasets. We compared EvoImp with well-established imputation methods documented in the literature. These datasets were systematically subjected to six missing value rates to simulate the Missing Completely At Random (MCAR) mechanism. The outcomes of these experiments were meticulously evaluated using five distinct classifiers. This comprehensive evaluation provides insights into the strengths and potential limitations of our EvoImp when applied to real-world multi-label classification scenarios. By addressing the challenges associated with missing data in this context, our work aims to advance multi-label classification and the broader field of data analysis.”

ED #18: Methods - Line 66-68: Revise/rewrite it. “… Recently, some new applications are being investigated in the areas of Computer 66 Vision, Natural Language Processing and Data Mining (DM), such as Video 67 Annotation, Legal Text Mining and User Profiling [21]. “

Thank you for your valuable feedback. We appreciate your attention to detail. We have revised the sentence in question as follows:

> “Recently, new applications have emerged in Computer Vision, Natural Language Processing, and Data Mining, including Video Annotation, Legal Text Mining, and User Profiling [30].”

References

[30] Liu W, Wang H, Shen X, Tsang IW. The emerging trends of multi-label learning. IEEE transactions on pattern analysis and machine intelligence. 2021 Oct 12;44(11):7955-74. DOI: 10.1109/T P AM I.2021.3119334

ED #19: Line 104-105 Revise the sentences…. "All these challenges have increased the complexity when dealing with MVs. On the 104 other side, it is not easy to find studies that related MLC and MV, as seen in [2,11,29]. In this scenario, we highlight a few studies addressing the problem of missing 106 labels [30,31], i.e., focusing on predicting an unknown label. "

Thank you for your feedback. We have revised the sentences in question:

> "All these challenges have amplified the complexity associated with handling MVs. Nevertheless, finding studies that relate MLC and MV is not straightforward, as demonstrated in [4, 8, 17].
>
> In this context, we emphasize a limited number of studies that specifically address the issue of missing labels [38, 39], which means focusing on predicting an unknown label.(...)"

ED #20: Line 110: change the word "relieve "

Thank you for your feedback. We changed the word "relieve" to "mitigate". The new period is:

> "For that, the authors use the definitions of multi-label neighborhood information entropy and multi-label neighborhood mutual information to mitigate the negative impact of missing labels."

ED #21: Line 111-112: merge two sentences: Cheng, Song & Qian [31] focus to dealing with missing labels using label correlations. 111 Therefore, the authors implements a two-level kernel extreme learning machine 112 autoencoder.

Thank you again. We merged these two sentences into:

> "Cheng, Song & Qian [39] focus on addressing missing labels by leveraging label correlations and implementing a two-level kernel extreme learning machine autoencoder."

ED #22: Line 113: Use the authors instead of "They "

Thank you for your feedback. We updated the manuscript according to your suggestion.

ED #23: Line 115-174: I suggest creating a table to describe the bio-inspired algorithms for missing value imputations. The current paragraphs are lengthy and very difficult to follow. The table will include columns such as
Previous bioinspired algorithms| Methods | strengths| limitation
Add it as a supplementary material, so that the readers can go back to the literature review.

As you recommended, we created a table summarizing the bio-inspired algorithms for missing value imputations. This table includes columns for Previous bioinspired algorithms, Methods, Strengths, and Limitations. Here is a table preview:

Table. Summary of Bio-Inspired Algorithms for Missing Value Imputations.

Previous bioinspired algorithms	Methods	Strengths	Limitations
Tran, Zang, and Andreae [1]	GPMI (Genetic Programming for Missing Value Imputation)	Applied a multiple imputation strategy<br>Performed missing value estimation using regression techniques	High computational complexity; The initial population is initialized randomly, which increases the search space
Shahzad, Rehman, and Ahmed [2]	Genetic Algorithm for Supervised Learning	Utilized Information Gain<br>Compared with multiple imputation approaches	The experimental setup considered only small datasets; and the comparison was made only against simple imputation methods
Lobato et. al [3]	MOGAImp (Multi-Objective Genetic Algorithm for Missing Data Imputation)	Adopted a multi-objective strategy, handling conflicting evaluation measures<br>Suitable for real-world applications<br>Deals with mixed-attribute datasets (categorical or continuous features).	The initial population is initialized randomly, which increases the search space
Lobato et al. [4]	MultImp (Multiple Imputations based on Genetic Algorithms)	Predicts multiple imputations in multi-label classification<br>Presents competitive performance	The experimental setup considered only small datasets; and the comparison was made only against KNNI and MC imputation methods

To make it more accessible to readers and, in line with your suggestion, we have added this table as supplementary material on our project's GitHub repository.

References

[1] Tran CT, Zhang M, Andreae P. Multiple imputation for missing data using genetic programming. In: Proceedings of the 2015 annual conference on genetic and evolutionary computation 2015 Jul 11 (pp. 583-590).

[2] Shahzad W, Rehman Q, Ahmed E. Missing Data Imputation using Genetic Algorithm for Supervised Learning. Int J Adv Comput Sci Appl. 2017;8.

[3] Lobato F, Sales C, Araujo I, Tadaiesky V, Dias L, Ramos L, Santana A. Multi-objective genetic algorithm for missing data imputation. Pattern Recognition Letters. 2015 Dec 15;68:126-31. DOI: 10.1016/j.patrec.2015.08.023

[4] Lobato FMF. Evolutionary strategies to optimize the treatment of missing data by multiple imputation data (in Portuguese). PhD Thesis, Federal University of Pará, 2016.

ED #24: Line 175: I suggest authors to develop a block diagram for each component of the GA structure:
Individual encoding and population initialization
Genetic Operators
Fitness Function
The Algorithm

Thank you for your suggestion. We have an illustration with each component of the Genetic Algorithm (GA) structure, included in the manuscript and also here for your convenience:

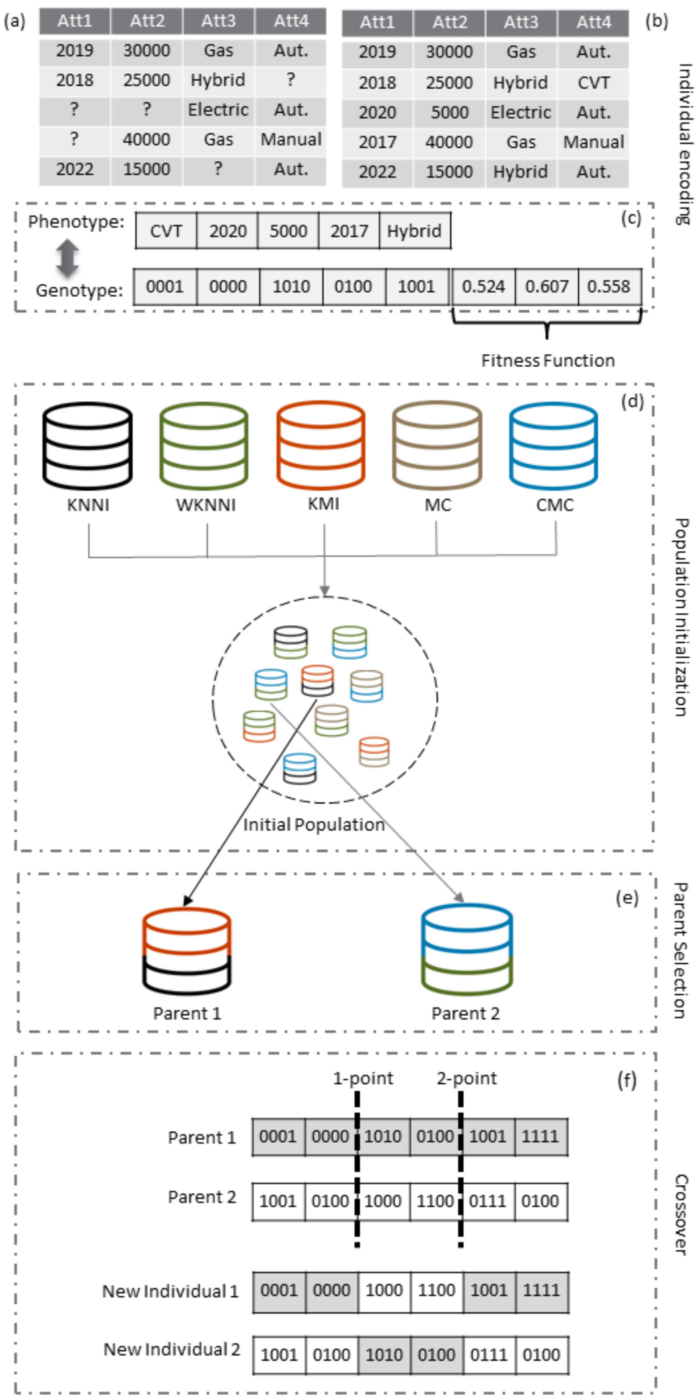

Fig 1. EvoImp's GA structure example. Toy example of a dataset with MV and how EvoImp's GA works with it. (a) Dataset with missing values; (b) A complete dataset with imputed data. (c) Phenotype: contains the values corresponding to the missing data space; Genotype: represents the genes in binary code and the values of the measurements used in the fitness function. (d) Illustration of how the initial population is initialized. (e) Random selection of parents for crossover. (f) Illustration of crossover being applied to the two selected individuals.

Additionally, we already have an algorithm's complete flowchart available on our project's GitHub repository (https://github.com/jacobjr/EvoImp) for further reference. To facilitate the review process, we present the flowchart below:

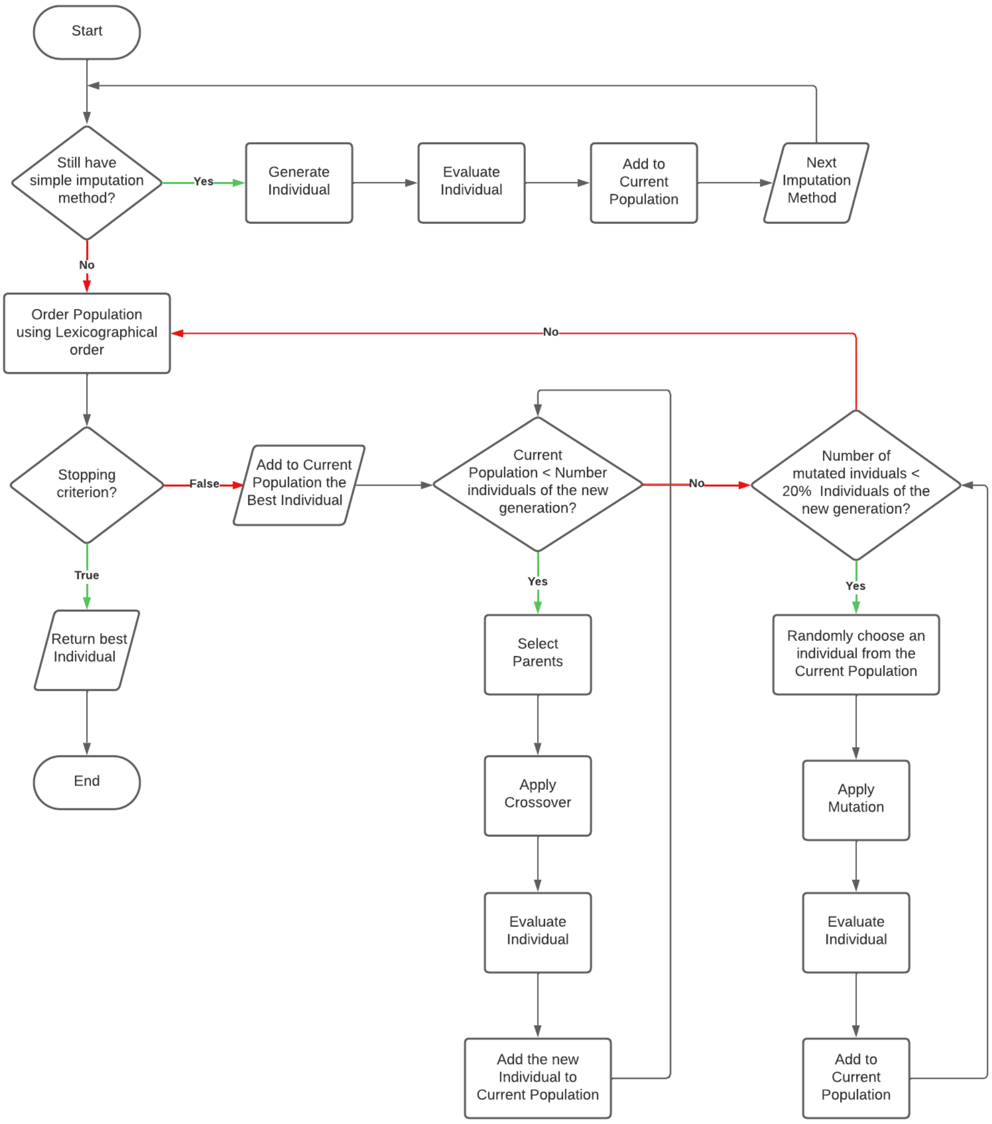

Fig 2. EvoImp's algorithm flowchart.

ED #25: Line 207- 212: Revise the sentences "Unlike MOGAImp [36], which uses random initialization of the initial population, the 207 proposed method relies on optimizing simple imputations through evolution to perform 208 multiple imputations. This strategy reduces the search space since it starts from a 209 priori solutions and represents the novelty compared to other methods. Furthermore, 210 this reduction allows for good use in scenarios where the computational cost for 211 calculating the objective function is sensitive, as in the case of multi-label classification. "

Thank you for your valuable feedback. We have revised the paragraph as suggested:

> "In contrast to MOGAImp [42], which employs random initialization of the initial population, the proposed method optimizes simple imputations through evolutionary processes to perform multiple imputations. This approach reduces the search space and introduces a novel method. This reduction in search space is particularly beneficial in scenarios where computational cost is critical in objective function calculations, such as multi-label classification."

ED #26: Line 341-345: These sentences are very confusing, needs to be revised "Regarding the simple imputation methods, the parameters recommended by [37] 341 were used. The mutation rate (MR) chosen is higher than the typical usage rates 342 because the starting point is not random. Therefore, considering that the initial 343 population is obtained by other methods, parameterization experiments demonstrated 344 that a higher MR yields better results, providing fast convergence. "

Once again, thank you for your valuable feedback. We have revised the paragraph as suggested. That's the new version:

> "Regarding the simple imputation methods, the parameters recommended by [7] were used. The mutation rate (MR) chosen is higher than the typical usage rates because the starting point is not random. Therefore, considering that the initial population is obtained by other methods, parameterization experiments demonstrated that a higher MR yields better results, providing fast convergence. (...)"

ED #27: Line 360-380: remove it to the supplementary file. Add one sentence in the main manuscript why we need to care about computational complexity for the proposed algorithm?

Based on your suggestion, we have removed the section titled 'Computational complexity of the proposed method' (lines 360-380) from the main manuscript. We have also added a paragraph emphasizing the significance of considering computational complexity for our proposed algorithm, as follows:

> "(...) The computational complexity is another crucial aspect to consider in implementing this proposed method. It plays a vital role in determining the feasibility and efficiency of applying bio-inspired techniques to solve optimization problems. Addressing this concern and reducing computational complexity enhances the algorithm's applicability and scalability. As a result, it makes it more suitable for handling larger datasets and complex optimization landscapes, particularly in multi-label classification tasks. More detailed information about EvoImp's computational complexity can be found in the supplementary materials on the project's GitHub repository."

ED #28: Table 5 and 6 7 8 9: Add footnotes by stating all the abbreviations of Db, KMI, KNNI MC< CMC, WKNNI ,EVoImp. What are these b, c, … ?? also explain what does it mean by uptick and downtick sign, what does the first column means- percentage of missingness? Then add it there

As you recommended, we added footnotes to Table 5, 6, 7, 8, and 9. Regarding the meaning of the uptick and downtick signs, in accordance with [39], we have included the following explanation:

> "The metrics (Exact Match (↑), Accuracy (↑), and Hamming Loss (↓)) are presented with these symbols, where (↑) indicates that higher values reflect better performance, and (↓) indicates that lower values represent better performance."

Reference

[39] Cheng Y, Song F, Qian K. Missing multi-label learning with non-equilibrium based on two-level autoencoder. Applied Intelligence. 2021 Oct 1:1-9. DOI:10.1007/s10489 − 020 − 02140 − 1.

Once again, we express our sincere gratitude for your meticulous review of our manuscript. We hope that our actions fulfill your expectations.

REVIEWER 1

R1#1. Author has revised my suggestion and I believe this article can be published.

We sincerely appreciate the reviewer's feedback and acknowledgment. We are delighted that the revisions have met the reviewer's expectations. Thank you for your valuable input, and we look forward to the opportunity to contribute to the journal.

<u>**REVIEWER 2**</u>

R2#1. The authors have provided a very thorough revision and have responded to all my comments.

Thank you for your support during these review rounds. Your pertinent observations were critical to improving the paper's quality. We are glad to learn that all your comments were properly answered.

R2#2. However, some grammatical errors or typos are shown indicatively below. Please check the whole manuscript again.
line 223: [41] provide → provides (or provided)
line 242: The table 2b → Table 2b
line 265: Exact Match: calculates → Exact Match calculates
line 330: 5, 10, 15, 20, 25, and 30% → 5%, 10%, …

Thank you for your careful reading. We have resolved all aspects raised. Also, we have carefully reviewed the entire manuscript to address any other grammatical errors or typos.

R2#3. In addition, a specific explanation of the mathematical notation needs to be given, such as in Eq. (3). Moreover, based on the description in the literature [54], it is recommended to redefine the mathematical expressions of Eqs. (5) and (6) to clarify the meaning of et al. Overall, the revisions have strengthened the paper by bringing improved clarity, and focus on contributions

Thank you for your pertinent observation. Given that one of the distinctions in this work lies in the utilization of lexicographical order, a thorough comprehension of these expressions becomes imperative. To facilitate this understanding, we have incorporated the following paragraph into the manuscript:

> As can be observed, the Eq. 5 shows $f_1 \prec_l f_2$, which means that f_1 is lexicographically less than f_2. This relationship is established when there exists an index k in in the range $[0, n_0) \cap \mathbb{N}$, such that $f_1^k < f_2^k$, indicating that the k-th component of f_1 is less than the k-th component of f_2. Additionally, the difference between f_1^k and f_2^k is greater than or equal to t. This ensures that the k-th components differ significantly by at least t. Finally, the absolute differences between corresponding components f_1^i and f_2^i should be less than t for all i less then k. In essence, this relation means that f_1 is superior to f_2 in terms of some objectives. The Eq. 6 determines equality in lexicographical order ($f_1 =_l f_2$). This occurs when the absolute differences between corresponding components f_1^i and f_2^i are all less than t for all i in the range $[0, n_0) \cap \mathbb{N}$. In other words, f_1 and f_2 are considered equal regarding their performance across objectives. Finally, the Eq. 7 presents $f_1 \preceq_l f_2$, which means that f_1 is either less than or equal to f_2 in lexicographical order. It combines the $\prec_l$ and $\preceq_l$ relations, indicating that f_1 is either better than or equal to f_2 in terms of the defined objectives.

We're pleased to hear that the revisions have enhanced the clarity and focus of the paper, emphasizing its contributions. If you have any further comments or suggestions, we would be happy to address them.
