# Supplementary material for: EvoImp: Multiple Imputation of Multi-label Classification data with a genetic algorithm
Source: PLoS One. 2024 Jan 19;19(1):e0297147. doi: 10.1371/journal.pone.0297147 (PMC10798481; doi:10.1371/journal.pone.0297147)
Supplement: Attachment — Submitted filename: comments.docx [file pone.0297147.s003.docx]

The authors have provided a very thorough revision and have responded to all my comments. However, some grammatical errors or typos are shown indicatively below. Please check the whole manuscript again.

- line 223: [41] provide → provides (or provided)
- line 242: The table 2b → Table 2b
- line 265: Exact Match: calculates → Exact Match calculates
- line 330: 5, 10, 15, 20, 25, and 30% → 5%, 10%, …

In addition, a specific explanation of the mathematical notation needs to be given, such as $\Delta$ in Eq. (3). Moreover, based on the description in the literature [54], it is recommended to redefine the mathematical expressions of Eqs. (5) and (6) to clarify the meaning of $n_{0}\text{, }f_{1}^{k}$ et al.

Overall, the revisions have strengthened the paper by bringing improved clarity, and focus on contributions.
